# Mechanical Plantar Foot Stimulation in Parkinson′s Disease: A Scoping Review

**DOI:** 10.3390/diseases8020012

**Published:** 2020-05-10

**Authors:** Lorenzo Brognara, Omar Cauli

**Affiliations:** 1 Department of Biomedical and Neuromotor Science, University of Bologna, Via Ugo Foscolo 7, 40123 Bologna, Italy; lorenzo.brognara2@unibo.it; 2 Frailty and Cognitive Impairment Organized Group (FROG), University of Valencia, 46010 Valencia, Spain; 3 Department of Nursing, University of Valencia, c/Jaume Roig s/n, 46010 Valencia, Spain

**Keywords:** gait, asymmetry, stride length, foot, rehabilitation, medical device, 3D printing

## Abstract

Background: Parkinson′s disease (PD) is the second most prevalent neurodegenerative disease in older individuals. Neurorehabilitation-based interventions such as those improving gait are crucial for a holistic approach and to limit falls. Several studies have recently shown that mechanical plantar foot stimulation is a beneficial intervention for improving gait impairment in PD patients. The objective of this scoping review is to evaluate the beneficial effects of this stimulation on gait parameters, and to analyse protocols of foot stimulation and other effects in non-motor symptoms. Relevant articles were searched in the Medline database using Pubmed and Scopus, using the primary search terms ‘foot stimulation’ OR ‘plantar stimulation’ AND ‘Parkinson’s disease*’. Several protocols have been used for mechanical plantar foot stimulation (ranging from medical devices to textured insoles). The gait parameters that have been shown to be improved are stride length and walking speed. The beneficial effects are achieved after both acute and repeated plantar foot stimulation. Beneficial effects are observed in other organs and systems, such as muscle activation, brain connectivity, cardiovascular control in the central nervous system, and the release of brain-derived neurotrophic factor and cortisol in blood added evidence about this intervention’s impact on brain function. Mechanical plantar foot stimulation is a safe and effective add-on treatment able for improving gait impairments in PD patients during the L-dopa off state. Randomized and controlled clinical trials to study its eventual potentiating effect with different pharmacotherapy regimens are warranted.

## 1. Introduction

Parkinson′s disease (PD), a chronic neurological disease characterized by degeneration of the dopaminergic neurons located in substantia nigra and alterations in other brain areas, and is the second most prevalent, chronic neurological disorder worldwide, with an increasing prevalence in the aging population [1]. Besides the symptomatic pharmacological treatment mainly consisting of dopaminergic drugs, neurorehabilitation represents an effective treatment for the management of PD [2,3,4,5,6]. Although the mainstay of treatment is pharmacological, non-pharmacological interventions are a vital part of a multidisciplinary approach for the optimal care of individuals with Parkinson’s disease [7,8]. Neuro-rehabilitative interventions have been used for some time in the treatment of PD but until recently, there has been less scientific evidence compared to pharmacological treatments to support the implementation of non-pharmacological interventions based on physiotherapy, occupational therapy, and speech and language therapy, which can have an impact on both motor and non-motor symptoms of PD. Among motor deficits observed in PD patients, gait disorders and postural instability have multifactorial causes related to alterations in straightening reflexes, rigidity and akinesia [9]. Gait disturbance occurs in both the early stages of the disease, when the gait is less swayed, turns are slower, and reductions in gait length are observed [10,11,12]. As the disease progresses, the gait becomes more unstable, gait freezing episodes occur, and falls are frequently reported [13,14].

Gait alterations impair the ability to perform simultaneous and sequential movements, and hypersensitivity to external stimuli that create blockages are disabling symptoms in PD patients, with adverse implications for mobility, independence in carrying out the activities of daily life and consequently a reduced health-related quality of life [15,16]. Although the main pharmacological therapies, i.e., dopaminomimetic drugs, are useful for the treatment of cardinal symptoms such as bradykinesia, stiffness, and tremor, their effect on gait deficits and postural instability is not as effective [9,17]. There is substantial evidence that the spatial characteristics of simple, straight-ahead gait is improved with levodopa and levodopa-enhancing drugs [17,18], but it is unclear whether more complex walking skills, such as gait initiation and gait adjustments, improve with dopaminergic treatment. The effects of dopaminergic treatment on stability measures of gait, such as spatial and temporal variability, are as yet inconclusive [17,19]. Some studies show that drug treatment can negatively affect aspects of gait and balance in individuals with Parkinson′s disease [17,20]. Almeida et al. [20] showed that the timing of walking to a stimulus is worse in patients in the L-dopa ON state compared to the patients who had withdrawn from the medication, perhaps because of L-dopa-induced dyskinesias. Almost 50% of individuals with PG display psychotic symptoms [21], and the concomitant use of neuroleptics can interfere and increase deficits in walking and balance [22].

Gait and balance control is determined by the integration of the somatosensory, the visual and the vestibular systems, and a reduction in the sensitivity of the foot is associated with a reduced control of compensatory stepping. Impairments in gait and balance are also characterized by alterations of the most important spatio-temporal gait parameters, such as gait velocity, cadence, stride time, and length, which are considered by patients to be the first and most worrying symptomatology. Alterations in the somatosensory system have been reported among non-motor symptoms in PD patients [23,24,25,26,27]. Increasing evidence suggests that plantar cutaneous mechanoreceptors appear to play an important role in regulating stepping during human gait, and that proprioceptive sensory input plays a vital part in the generation and coordination of movements; alterations in the peripheral afferent inputs and/or their central processing alterations influence motor disability in patients with PD.

Reinforcement of automatic movements through sensory-evoked phasic release of dopamine is hypothesized as being crucial to the maintenance of automatic movements [28,29,30,31,32]. Quattrocchi et al. indicate that a controlled pressure mechanical stimulation at two specific points of both feet increases brain functional connectivity of the primary sensory-motor cortices with the left superior parietal lobule and the left lateral occipital cortex (an important area involved in sensory-motor and visuo-spatial integration and processing) [33,34].

Over the last decade, several reports have pinpointed the importance of peripheral sensory feedback at the level of plantar feet and have demonstrated beneficial effects in improving clinical symptoms in PD related to gait and balance impairments.

The aims of the review were:

- To analyze the protocol of plantar foot stimulation performed in PD patients.
- To analyze the resulting effects on spatiotemporal gait parameters induced by mechanical plantar foot stimulation.
- To analyze any eventual beneficial effect on non-motor-symptoms induced by mechanical plantar foot stimulation.

## 2. Methods

We chose to conduct a scoping review in order to map the existing literature. A scoping review differs from a systematic review, as instead of assessing the literature to provide an answer to a specific question, it systematically surveys the literature, quantitatively synthesizes what has been produced, and summarizes the gaps in the literature in a specific field of research. As the area of mechanical plantar foot stimulation biomarkers and gait impairment in Parkinson’s disease is relatively new, there are to date no systematic reviews on this topic. A scoping review was therefore undertaken to examine the extent, range, and nature of research activity in this area, to highlight areas where more research is needed. The methodology does not significantly differ from a systematic review, although publications are not excluded based on study design, and quality assessments of each study are not undertaken. A scoping review typically precedes a systematic review in ordre to survey the literature and identify relevant research questions to be answered by a later systematic review. Furthermore, a scoping review does not aim to aggregate findings using meta-analysis, but instead maps the literature to date to identify themes, trends, and gaps. We outlined the duration and type of treatments, average pressure applied, clinical features assessment period and instruments used to assess the effect of therapy on gait spatio-temporal parameters, other motor and non-motor symptoms and physiological alterations. To that end, we analyzed every original article in the PubMed/Medline and Scopus electronic bibliographic databases, published until February 2020, which met the following inclusion criteria: (1) full text in English; (2) primary articles only; and (3) presentation of identifiable data measuring gait and balance parameters. When determining which articles to include, we analyzed their title and abstract, and the full text was then retrieved for articles that met the inclusion criteria. Finally, the reference lists of all the relevant articles were manually cross-referenced in order to identify any additional articles. The primary search terms used were ‘foot stimulation’ OR ‘plantar stimulation’ AND ‘Parkinson’s disease*’. We excluded conference proceedings and articles that did not assess gait, balance, or other symptoms in PD patients. 

## 3. Results

The articles were searched in common literature databases (PubMed and Scopus). We evaluated 109 abstracts in Scopus and after removal of duplicates and the selection process, our search selected 11 articles fulfilling the inclusion criteria. We reported the protocol of plantar foot stimulation performed in PD patients, the effects of plantar foot stimulation in different parameters related to gait impairment (the descriptions of these parameters are shown in Table 1) and non-motor symptoms and other effects induced by mechanical plantar foot stimulation. The corresponding anatomical position of stimulation with respect to the foot and the protocol features were also reported.

### 3.1. Protocols of Plantar Foot Stimulation and Sites of Stimulation

A summary of the protocols used and the anatomical areas is presented in Table 2. The most used protocol and sites of stimulation on feet consist in stimulating four target areas (two per foot, at the head of the big toe and the base of the first metatarsal bone) applying a pressure of 0.3–0.9 N/mm^2^ for 2 min, reported in six sham-controlled studies (six out of 11). Various stimulation protocols have been tested in terms of the instrument and pressure applied for the stimulation. Plantar foot stimulation using the Gondola^TM^ device enables the exactly applied average pressure of 0.3–0.9 N/mm^2^ to be reported. Only one study (Barbic et al. [54]) using procedures other than the Gondola device reported the value of pressure stimulation at the level of stimulated areas of the plantar foot achieving a mean pressure of 0.6 (aprox) kg/mm^2^ measured by a steel stick with a smooth, 2-mm-diameter tip connected to a dynamometer. The magnitude of the mechanical pressure triggering the reflex withdrawal was assumed to be the patient’s individual pressure point and indeed, it was subsequently used during the effective stimulation procedure [54]. The Gondola device is composed of two units, worn on the patient’s feet for the short duration of the treatment (less than two minutes). In studies performed with the Gondola [55] device, patients must be laying on a bed while receiving the treatment. The stimulation lasts less than two minutes, while the overall time required by the treatment from beginning to end is approximately ten minutes. The studies using insoles to stimulate plantar foot used a static protocol of stimulation prior to analysis of the gait [54,56]; or used textured insole [57,58,59]. A variety of stimulation periods were found with the other procedures, ranging from a few seconds [54], minutes [56], and even days [59]. As regards the anatomical location of mechanical stimulation on plantar foot, most studies stimulated the head of the big toe and the base of the first metatarsal bone. Jerkins et al. [57] performed plantar stimulation using a facilitatory insole with a ridge positioned at the lateral aspects of the plantar surface of the foot. In contrast, the studies by Qui et al. [58] and Lirani-Silva et al. [59] used textured insoles to achieve the mechanical stimulation of the plantar feet. The clinical features of PD patients enrolled in these studies are classified in the Hoehn and Yahr staging scale as level 2–3 in most of the studies. The studies by Pagnussat et al. [60] and Brognara et al. [56] also included patients in stage 4 (e.g., severe disability; still able to walk or stand unassisted). In the study of Qiu et al., the mean Hoehn and Yahr staging scale was 1.4, and as such so the clinical features of these patients were milder compared to the rest of the analysed studies. Motor impairment was assessed in all studies using the last version of the motor section of the Unified Parkinson’s Disease Rating Scale (UPDRS III) and scores ranged from a mean score of 24 and over. Both scales were evaluated during the OFF-levodopa phase (at least 12 h after the last medication dose). As for the experimental procedure to stimulate the plantar foot, all studies except one (Jenkins et al. [57]) performed the measurement during the OFF-levodopa phase, whereas the latter study tested the participants 1–2 h after dosage with the medication, at their self-selected best “ON” time.

### 3.2. Effects of Plantar Foot Stimulation on Various Parameters Related to Gait Impairment

The effects of mechanical plantar foot stimulation on spatiotemporal gait parameters vary among the studies (Table 3). Different numbers of instruments were also used to assess the clinical features. Gait speed and stride length are the most common parameters with improvements, reported in six studies. The spatiotemporal gait parameters were assessed in a three-dimensional movement analysis laboratory in five out of 11 cases. Other instruments used in the assessment of gait parameters included inertial measurement units (IMUs) and the GAITrite^TM^ mat (a smooth surface mat equipped with 16,000 sensors on a portable pressure sensitive walkway providing temporal spatial gait analysis). The beneficial effects of mechanical plantar foot stimulation on several gait parameters were reported in all the studies that evaluated them (N = 7). The most common beneficial effects of mechanical plantar stimulation reported were gait velocity (reported in seven studies: [55,57,60,62,63,64,65]), stride length (reported in 6 studies: [55,56,59,60,63,64]) and step length (reported in five studies [57,61,62,64,65]) (Table 3). However other parameters less extensively investigated in the gait analysis also improved after plantar foot stimulation, including step variability (reported in two studies [57,62]), asymmetry and pitch contact (reported in 1 study [56]) (Table 3). An important issue for understanding how this intervention acts is the reporting of gait parameters that did not improve walking speed [56,59].

The effect on posture parameters such as center of pressure, sway area and standing balance was evaluated using a force plate in two studies (Prusch et al. [61], Qiu F et al. [58]), in which gait parameters were not evaluated. Six studies reported an evaluation of clinical features performed before (baseline) and at the end of plantar foot stimulation. The effect of mechanical plantar foot stimulation on posture and balance was reported in two studies, but no analysis of gait parameters was performed [58,61]. The center of pressure parameters such as pressure area, sway range and mean velocity were evaluated. None of these parameters changed significantly after stimulation with the Gondola device [61]. In contrast, using plantar foot stimulation by wearing a textured insole, Qiu et al. [58] observed a reduction in the medial-lateral sway and medial-lateral sway in the PD group. 

### 3.3. Other Effects of Mechanical Plantar Foot Stimulation

Mechanical plantar foot stimulation showed other effects besides improvement in gait parameters. Plantar foot stimulation improved sensory perception at the foot level. Lirani-Silva et al. used the Semmes–Weinstein monofilament to demonstrate that the continuous use of textured insoles improved plantar sensation, and the benefits persisted after the follow-up period [59]. The beneficial effects of mechanical plantar foot stimulation on gait showed parallel improvements in other organs and tissues, such as muscle activity [57], autonomic control of heart rates and blood pressure [65] and the circulating levels of the neurotrophin brain derived neurotrophic factor (BDNF) and cortisol in blood (Pagnussat et al. [60]). Jenkins et al. [57] demonstrated wearing a facilitatory (ribbed) insole positioned at the lateral aspects of the plantar surface improved not only gait parameters (gait velocity, step length, single-limb support time, and step-to-step variability) but also muscle activation patterns. Muscle contraction in gait patterns was evaluated by surface electromyography. The use of this insole normalized the muscle activation timing of the tibialis anterior and gastrocnemius muscles at the point of ground contact among individuals with PD. Alterations in cardiovascular autonomic control resulting in orthostatic hypotension have been described in 10%–40% of patients with PD (Palma and Kaufmann 2020). Orthostatic hypotension may worsen motor disability, and represents a risk factor for falls in PD patients [66]. Barbic et al. evaluated whether mechanical activation of feet sensory afferents could improve gait and modify the response of cardiovascular autonomic control to stressors (supine or 75° head-up tilt). The patients enrolled in the study were characterized at baseline by considerable alterations in cardiovascular autonomic control, namely by a blunted capability to activate the sympathetic nervous system controlling heart rate and arterial vessels in an upright position. Twenty-four hours after plantar stimulation, an increase in the increased cardiac and vascular sympathetic modulation during upright position was observed compared to the baseline (before plantar stimulation) [54,65]. The authors suggested that the effect on cardiovascular autonomic control might be due to the activation of a tactile and/or nociceptive afferent pathway from the feet, projecting to the medulla oblongata where the centers of cardiovascular control are located [67]. Pagnussat et al. showed that the repeated automated mechanical peripheral stimulation (eight treatment sessions) with Gondola devices can increase the levels in blood of the neurotrophin brain derived neurotrophic factor (BDNF) and reduce the level of the stress hormone cortisol. In addition, a significant correlation was observed between the rise in BDNF levels after plantar stimulation and improvements in gait speed, stride length and TUG performance. One of the roles of BDNF is to maintain the survival and the differentiation of dopaminergic neurons [68,69] and to reduce the apoptosis-mediated cell death and degeneration of dopaminergic neurons [70]. Meanwhile, stress in humans increases the concentration of cortisol in blood [71,72]. At excessive concentrations, cortisol can suppress both the synthesis and the release of BDNF, and is inversely correlated with BDNF levels [60,69,73,74]. In previous studies, cortisol levels were found to be high in PD patients compared to subjects in the healthy control group [73,75]. The authors suggest that these changes at the levels of BDNF and cortisol promote improved brain connectivity and increase the release of dopamine from spared dopamine nerve terminals [34].

## 4. Discussion

Despite PD being considered a neurodegenerative disease with primary alteration of the motor system, e.g., basal ganglia and its connectivity with the cortex, it has recently been suggested that sensory abnormalities play a significant role in motor alterations. Although it is considered that these alterations at the level of the sensory system affect the feedback of the motor system, thus causing observable motor deficits, it is not known whether the sensory deficits are due to a reduction in the function of the peripheral sensory receptors, or to some subtype of receptors, or simply due to a deterioration of the central sensory-motor integration or a combination of both mechanisms. In fact, PD patients display deficits in both sensorimotor integration [76,77] and peripheral sensory function [78,79]. Several reports have also demonstrated in individuals without neurological diseases that a decreased plantar cutaneous sensation achieved impair static and dynamic balance control [80,81]. Conversely, sensory stimulation of the plantar cutaneous surface with textured insoles has been shown to improve balance and gait in older individuals with a history of falls [82]. PD patients that display severe gait impairments can particularly benefit from interventions based on mechanical stimulation of the plantar foot, thereby improving gait parameters and in turn limiting the adverse outcomes related to that impairment. Different methods of mechanical plantar foot sensory stimulation have been studied, including insoles with a raised ridge located at the foot’s perimeter, mechanical pressure on the sole of the foot, and vibratory insoles [83]. Several studies have tested automated mechanical peripheral stimulation (patented as Gondola^TM^) to facilitate the stimulation procedure through an electric motor that activates metallic stimulators with a diameter of 2 mm. All of these results support plantar foot stimulation being able to enhance some spatio-temporal gait parameters such as gait speed, step and stride length in individuals with Parkinson′s disease evaluated at baseline (pre), after the first session (post 1st), after the fourth session (post 4th) and at the end of the 8th session (after four weeks). Emerging gait parameters to study the effect of plantar stimulation such as the study of pitch contact are promising. This parameter, representing the angle between the ground and the foot during heel contact appeared to be improved by acute stimulation of the plantar foot [56]. This parameter could be a useful indicator of the power of contraction of the anterior tibia muscle and the range of motion of the ankle during walking. Ankle dorsiflexion is a crucial factor in starting the gait by allowing the foot to strike the ground in a position that facilitates movement of the sagittal plane during the heel strike; progressive momentum is thereby preserved. Confirming this, the use of a textured insole significantly increases the single-limb support time, by normalizing the muscle activation sequence of the tibialis anterior at the time of initial ground contact [57]. There is a lack of knowledge about the duration of therapy or daily stimulation time that may be a key component in possibly explaining the different effects in the gait spatio-temporal parameters reported. This scoping review is the first to compare the effect of different mechanical plantar foot stimulation techniques and devices on clinically relevant aspects of gait in individuals with Parkinson’s disease. An important issue for understanding the different effects is to analyze the reports of studies in which some features did not improve, such as walking velocity in two studies [56,59]. Part of these discrepancies among the studies analyzed could be attributed to the variation of the clinical stage and the rate of the motor impairment among the participants enrolled in the study. Accordingly, future trials should compare the effects of mechanical plantar foot stimulation in patients with different severity of the diseases. The majority of studies, except for one, evaluated plantar stimulation during the off phase of L-dopa (12 h from the latest drug administration), and the possible potentiating effect of dopaminergic therapy needs further studies. The drugs used to treat PD symptoms and comorbid symptoms can negatively affect some aspects of gait and balance impairment and the review of pharmacotherapy, e.g., risks/benefits analysis is crucial for improving the care of PD patients during the progression of the disease over time [17,21]. Future studies involving large samples of PD patients with different pharmacotherapy regimens should be carried out in order to observe possible synergies between mechanical foot stimulation and different gait parameters. Consensus on the stimulation and assessment protocol is still lacking in terms of the instruments applied, duration of treatment and assessment period for clinical outcomes. Outcome measures should include patient perceptions of the cost-effectiveness and long-term efficacy of the treatment.

In terms of the effects on posture/balance parameters, Qiu et al. demonstrated that stimulation performed by a textured insole can reduce medial-lateral sway and medial-lateral sway standard deviation in individuals with PD, improving postural stability. In contrast, Prusch et al. showed that the Gondola^TM^ device has no positive effect in terms of improving static postural control in individuals with PD. Besides these contrasting results, further studies should investigate the effects on static and dynamic balance control in individuals with PD, and also compare the different instruments used. The studies that tested the effects of textured insoles also showed improvements in stride length and balance control, but in the future a decision regarding the different type of stimulation insoles should be based upon the best evidence, outcome measures and long-term effectiveness in terms of both the frequency of stimulation, insole replacement and a comparison between prefabricated and custom-made devices. The fact is that mechanical plantar foot stimulation treatments increased BDNF levels and lowers cortisol concentration in blood which can impact positively mental functions [84], thus, improving further, the quality of life for patients. The positive effects of plantar foot stimulation on orthostatic hypotension may add an important benefit to reduce the risk of falls in these patients.

## 5. Conclusions

The review of the current evidence shows that mechanical foot sensory stimulation is a valid neuro-rehabilitative intervention for improving clinical symptoms in PD related to gait impairments. Evidence for the effects of these interventions on reducing falls in PD patients is still lacking, and longitudinal studies are necessary. The analysis of patient subgroups who benefit most from these interventions and the interaction with pharmacotherapy warrant future clinical trials.

## Figures and Tables

**Table 1:** Table showing all spatiotemporal parameters of interest [35,36,37,38,39,40,41,42,43,44,45,46,47,48,49,50,51,52,53].

Type of Parameters Related to Gait Impairment	Measurement	Alteration in PD
**Variability** (s) Pre > post (the index must decrease compared with Pre-MPS)	Standard deviation of stride duration	PD gait includes a loss of consistency in the ability to produce a steady gait rhythm, resulting in higher stride-to stride variability.
**Asymmetry** (%) Pre > post (the index must decrease compared with Pre-MPS)	Difference in speed between right and left side	Changes in PD gait include increased left right gait asymmetry and diminished left-right bilateral coordination.
**Gait speed** (m/s) Pre < post (the index must increase compared with Pre-MPS)	Ratio between the length and duration of the stride	Speed is often considered a clinical vital sign in PD gait and declines much faster. Slow gait speed is significantly related to clinical ratings of disease.
**Stride length** (m) Pre < post (the index must increase compared with Pre-MPS)	Distance between successive ground contacts of the same foot (opposite foot regarding Step length)	Defective scaling of stride length underlies gait disturbance in PD
**Cadence** (step/min) Pre = post (No significant interactions compared with Pre-MPS)	Number of steps per minute (stride frequency).	Gait in PD is associated with shorter stride lengths, and greater significance in cadence was found in patients with PD compared with a control group
**Stride duration** (s) Pre > post (the index must decrease compared with Pre-MPS)	Time elapsed between the first contact of two consecutive footsteps of the same foot	Stride duration depends on the level of gait speed; a significant association with high stride duration was demonstrated at low speeds.
**Swing phase** (%) Pre < post (the index must increase compared with Pre-MPS)	The swing phase is the part of the gait cycle during which the reference foot is not in contact with the ground and swings in the air. It constitutes about 40% of gait cycle.	Greater significance in swing phase and in swing duration was found in patients with PD compared with a control group
Single support and double support Pre > post (the index must decrease compared with Pre-MPS)	Double support (2 times 10%); when only one is in the support phase. Single support (40%), the second is then in the oscillating phase.	Stance phase was not significantly different in patients compared with healthy subjects. Patients with PD spent more time (compared with a control group) in double support phase of gait, but these changes were not significant
Pitch Contact (°) Pre < post (the index must increase compared with Pre-MPS)	Contact angle between the foot and the ground during the first step.	Patients with PD have reduced pitch contact due to an inadequate heel clearance and may have increased fall risk.

**Table 2:** Characteristics of protocol for mechanical plantar foot stimulation.

Reference	Experimental Group (N = Mean Age, Sex)	Control Group (Type, N = Mean Age, Gender Distribution % of Female)	Anatomical Areas of Plantar Foot Stimulation and Stimulation Instrument	Duration and Type of Treatment (Number of Times/Days if Repeated Stimulation Was Done)
Brognara et al. (2020) [56]	N = 12; M/F = 6/6	N = 12; M/F = 4/8	3D custom insole with the mechanical stimulation with two blunted cone size 5 × 2 × 7 mm under the distal phalanx of the big toe and underneath the head of the first metatarsal joint of both feet.	Acute (one day) treatment consisted of staying in the orthostatic position (stable standing), wearing insoles, which administer the stimulations, for 5 min. Gait was assessed pre- and post-stimulation without wearing insole.
Prusch et al. (2018) [61]	AMPS group: N = 16; M/F = 13/3	AMPS sham group: N = 17; M/F = 11/6	AMPS was administered using Gondola^TM^.	Subjects with PD underwent eight sessions of real or placebo AMPS, once every 3–4 days. The total experimental period lasted 4 weeks
Pagnussat et al. (2018) [60]	AMPS N = 16; M/F = 13/3	AMPS** sham group: N = 16; M/F = 10/6 and 12 healthy subjects (4M/8F)	AMPS was administered using Gondola^TM^	Subjects with PD underwent eight sessions of real or placebo AMPS, once every 3–4 days. The total experimental period lasted 4 weeks
Kleiner et al. (2018) [62]	N = 15; M/F = 12/3	N = 15; M/F = 9/6	AMPS was administered using Gondola^TM^	2 treatment sessions a week for 4 consecutive weeks.
Pinto et al. (2018) [55]	N = 15; M/F = 12/3	N = 15; M/F = 9/6 and 14 healthy subjects (9M/5F)	AMPS was administered using Gondola^TM^	Subjects with PD underwent eight sessions of real or placebo AMPS, once every 3–4 days. The total experimental period lasted 4 weeks.
Lirani-Silva et al. (2017) [59]	N = 10; M/F = not reported	N = 9; M/F = not reported	Distal phalanx of the hallux, heads of metatarsophalangel joints and heel using textured insoles with half-sphere elevations (9 mm diameter).	Participants wore group-specific insoles (textured/conventional) for one week and conventional insoles in the following week.
Kleiner (2015) [63]	N = 35 Sex distribution not reported	N = 35; M/F = not reported Healthy adults	AMPS was administered using Gondola^TM^	The treatment consists in 4 cycles; one cycle includes a stimulation of the 4 target areas requiring 24 s. Gait was assessed pre- and post- stimulation.
Stocchi (2015) [64]	N = 18 Sex distribution not reported	N = 15 healthy individuals	AMPS was administered using Gondola^TM^	Patients were treated with AMPS six times once every 4 days.
Barbic (2014) [54]	N = 8; M/F = 4/4	N = 8; M/F = 4/4; Sham group	2 points for each foot (at the tip of the hallux and first metatarsal joint. Sham stimulation was performed at the level of two plantar skin sites other than those selected for effective stimulation).	Stimulation was applied for 6 s, repeated four times in each subject in one day.
Qiu (2013) [58]	N = 20; M/F = 13/7	N = 20; M/F = 13/7; healthy	The textured insoles used in this study were 1.5 mm thick and constructed using soft insole material. The textured surface comprised granulations measuring 5.0 mm in diameter and 3.1 mm in height. The texture was located around the lateral perimeter of the insole and around the heel of the foot.	Patients were evaluated in three different footwear conditions: (1) barefoot; (2) wearing smooth insoles and (3) wearing textured insoles.
Jenkins (2009) [57]	N = 40; M/F = 24/16	controls without neurological disease N = 40; M/F = 15/25	On lateral aspects of the plantar surface using an insole with a ridge positioned at the lateral aspects of the plantar surface.	Subjects used an insole during gait for analysis only.

Abbreviations: MPS: Mechanical peripheral stimulation. AMPS: Automated Mechanical Peripheral Stimulation using Gondola^TM^ consisting of foot supports with electric motors that activate metallic stimulators with a diameter of 2 mm. Pressure is applied in four target areas (two per foot, at the head of the big toe and the base of the first metatarsal bone). Stimulation was applied in a range of 0.3–0.9 N/mm^2^ for 2 min.

**Table 3:** Characteristics of the protocol for the analysis and effect on spatio-temporal gait and postural control parameters.

Reference	Protocol for the Analysis of Spatio-Temporal Gait Parameters	Effect of MPS on Spatio-Temporal Gait and Postural Control Parameters
Brognara et al. (2020) [56]	Gait analysis was assessed by wearing portable inertial sensors (pre- and post-evaluation).	Improved gait variability, gait asymmetry, stride length and pitch contact. However, no effects in speed, cadence, stride duration, swing phase, single and double support.
Prusch et al. (2018) [61]	Assessment was performed before (baseline) and at the end of the 8th session (after 4 weeks) of stimulation using a single force platform.	No significant improvement in static postural control
Pagnussat et al. (2018) [60]	Assessment was performed before (baseline) and at the end of the 8th session (after 4 weeks) using an inertial measurement unit. Time up and go (TUG) performance was also assessed.	Improvements in velocity and stride length (increased). Improvement in TUG time (improved performance).
Kleiner et al. (2018) [62]	Gait was assessed at baseline and after the first, fourth and eight treatment session in a movement analysis laboratory with a gait analysis using a three-dimensional optoelectronic system.	Improvements in gait asymmetry, step length, step time, gait velocity, step length standard deviation and step time standard deviation. The AMPS group showed an improvement in gait variability after 8 stimulation sessions.
Pinto et al. (2018) [55]	Assessment was performed before (baseline), after the first session (POST 1st), after the fourth session (POST 4th), and after the eighth session (POST 8th) using a three-dimensional motion analysis system.	Improvements in stride length, step length and gait speed.
Lirani-Silva et al. (2017) [59]	Gait was assessed at three points in time using an optoelectronic tridimensional system at a self-selected speed on an 8-m long pathway without the textured insoles: (1) before (pre-test) (2) after one week wearing the group-specific insoles (post-test) (3) after one week wearing conventional insoles (follow-up).	The treatment group participants showed a greater stride length Post-test compared to Pre-test.
Kleiner et al. (2015) [63]	Gait analysis was assessed by an inertial measurement unit (IMU).	Improvements in stride length, gait velocity, and gait propulsion.
Stocchi et al. (2015) [64]	Gait parameters were assessed in a movement analysis laboratory using a three-dimensional optoelectronic system after the first intervention, after the sixth intervention, 48 h after the sixth intervention, and 10 days after the end of the treatment.	Improvement in velocity, step and stride length, and walking stability.
Barbic et al. (2014) [65]	Assessment was performed before (baseline) and 24 h after stimulation using a three-dimensional movement analysis.	Improvements in velocity, step length, gait symmetry (partial rebalancing in half of the patients, rotation velocity and rotation steps).
Qiu et al. (2013) [58]	Participants performed standing tests on two different surfaces (firm and foam), under three footwear conditions. Standing balance was evaluated using a force plate.	Textured insole reduced medial-lateral sway and medial-lateral sway standard deviation in the PD group.
Jenkins et al. (2009) [57]	Assessment was performed comparing the performance using the stimulation insoles and using a conventional (flat) insole (instruments used: GAITRite)	Improvements in gait velocity, step length, single-limb support time, and step-to-step variability of step length.
